# Influence of Preheating on the Microstructure Evolution of Laser Re-Melting Thermal Barrier Coatings/Ni-Based Single Crystal Superalloy Multilayer System

**DOI:** 10.3390/ma12193088

**Published:** 2019-09-22

**Authors:** Zhengjie Fan, Wenqiang Duan, Xiaofeng Zhang, Xuesong Mei, Wenjun Wang, Jianlei Cui

**Affiliations:** 1 State Key Laboratory for Manufacturing Systems Engineering, Xi’an Jiaotong University, Xi’an 710049, China; wenqiangduan@xjtu.edu.cn (W.D.); xsmei@xjtu.edu.cn (X.M.); wenjunwang@xjtu.edu.cn (W.W.); cjlxjtu@xjtu.edu.cn (J.C.); 2 Guangdong Institute of New Materials, Guangzhou 510650, China; zxf200808@126.com

**Keywords:** laser processing, TBCs, thermal damage, single crystal superalloy, induction heating

## Abstract

Laser surface re-melting (LSR) is a well-known method to improve the properties of atmospheric plasma-spraying thermal barrier coatings (APS TBCs) by eliminating the voids, incompletely melted particles and layered-structure. Laser energy density should be carefully selected to reduce the exposed thermal damage of the underlying single crystal (SX) matrix. Therefore, the purpose of this paper was to identify the effect of introducing induction heating to laser modifying of APS TBCs coated on Ni-based SX superalloy. The results indicated that the preheating of the substrate can lower the laser energy threshold that is required for continuously re-melting the coating. It proved that, in LSR processing of a APS TBCs/ SX matrix multilayer system, the combined method of adopting the low laser energy and preheating at elevated temperature is an effective means of minimizing the cracking susceptibility of top ceramic coating, resulting from decreasing the mismatch strain between the re-melted layer and residual APS TBCs, which can significantly improve the segmented crack condition in terms of crack dimension and crack density. Moreover, this combined method can remarkably lower heat input into an SX matrix and correspondingly the interface stored energy induced by pulsed laser thermal shock, which can effectively lower the tendency for surface recrystallization after the subsequent heat treatment.

## 1. Introduction

Thermal barrier coatings (TBCs) are commonly applied to the surface of turbine blades to protect them from high temperature exposure and eventual damage. TBCs systems consist of a thermally-insulating ceramic top coating, a thin thermally-grown oxide layer, and an aluminium-rich metallic bond coating [1,2], which are used as gas turbine components to prevent the underlying materials from exposure to extreme environments. The typical TBCs material comprise 7–8 wt % Y_2_O_3_–stabilized zirconia (YSZ) due to significant improvements in durability and adequate fracture toughness [3]. Air plasma spraying (APS); electron beam physical vapor deposition (EB-PVD); and modified plasma-spraying methods, such as suspension plasma spraying (SPS), are currently used widely for the deposition of TBCs [4,5,6]. Plasma spraying has become one of the most common manufacturing methods to fabricate TBCs [7]. The TBCs will degrade in long-term serve at extremely environment, such as at high heat flux, thermal cycling and hot gases environment contained sulfur, sodium and vanadium contaminants [8]. However, manufacturing TBCs which present both superior hot corrosion resistance and high thermal cycle lifetime is still a real challenge.

The laser surface re-melting (LSR) technology is currently recognized as a most promising method to enhance the high temperature performance of TBCs owing to their high process selectivity, localized heating and fewer restrictions of materials [9,10,11,12]. The LSR can seal the porosity, eliminate the lamellar structures, and inter-splat voids and microcracks in atmospheric plasma-spraying (APS) TBCs to produce a fully dense and smooth re-melted top layer [13,14,15,16,17]. The high cooling rate and high solidification rate of the LSR process can develop a fine homogeneous microstructure and the columnar grains microstructure, which can enhance strain tolerance and fracture toughness of the coatings [18,19,20]. Recent studies indicated that the LSR can considerably improve the hot corrosion resistance of the APS TBCs mainly owing to the sealing of porosity and reduction of the specific area exposed to molten salts [21,22,23].

Besides the formation of the dense re-melted layer, LSR can develop vertical segmented cracks and horizontal cracks, due to the shrinkage and relaxation of residual stresses in the process of non-uniform and rapid cooling (the cooling rate up to range of 10^3^~10^4^ °C S^−1^) [24,25,26,27]. The vertical segmented cracks are widely considered to be helpful for accommodating the oxidation stress and mismatch stress. The segmented cracks play a major role in TBC lifetime extension and increasing the lifetime of as-sprayed TBCs by about double- to six-fold [11,21,26,27]. However, considering the horizontal cracks aspect, they can accelerate the coating spallation [18,25,28,29]. Therefore, it is necessary to avoid the horizontal cracks which occur in laser re-melted TBCs. Nevertheless, the segmented cracks become deeper and more severe with increasing input energy. Induced by the defects (i.e., microcrack, voids, unmelt powder) in porous APS coating, the cracks tend to deviate from the vertical direction to horizontal direction [18,30,31], which accordingly causes a risk of TBCs spallation when subjected to thermal cycle shock [20]. Moreover, in our previous work, we found the molten salts infiltrate into coatings preferentially through coarse segmented crack and react with the stabilizer (Y_2_O_3_), leading to a reduction in strain accommodation of the TBCs [32]. The introduction of overmuch macroscopic vertical segmented crack can also cause an elevated thermal diffusivity problem [33]. Therefore, to achieve an overall modified performance of the TBCs, the segmented cracks should be reasonably controlled in terms of their depth (i.e., less than the thickness of re-melted layer), width and density.

Another concern is probable thermal damage of the underlying metallic matrix using high energy laser re-melting TBCs. However, limited attention has been given to microstructural evolution of the substrate in laser re-melting TBCs. As research has indicated, the intensive laser heating, resulting from unreasonable laser parameter selection, can lower the mechanical properties, even the unacceptable dilution of the underlying metallic matrix [34]. More importantly, the concern of thermal damage of the matrix is particularly sharp involving the single crystal (SX) superalloy components, as surface primary recrystallization, creep and mechanical degradation can most likely arise under high temperature and high-stress conditions [35,36,37]. Nickel-based SX superalloys have been widely used as a turbine blade material due to their extremely excellent elevated temperature mechanical properties due to almost complete elimination of all grain boundaries [37,38]. However, the manufacturing process may introduce the plastic deformation and surface recrystallization may occur during the subsequent heat treatment [39,40]. The recrystallization can cause significant creep and mechanical properties degradation because of the removal of grain boundary-strengthening elements i.e., C, B and Zr [35]. It was reported that the residual stresses formed during coatings preparation can affect interfacial creep and thermal growth oxide (TGO) growth mechanisms and introduce cellular recrystallization under exposure to a high temperature [41].

Preheating has revealed a high potential for the improvement of laser re-melted TBC properties by reducing the cooling rate, smoothing the temperature fluctuation of molten pool [25,42]. It was indicated that the preheated coatings are subject to less quenching and correspondingly less shrinkage as well as the low-level residual stress upon cooling, thus forming less severe cracks, significantly reducing the crack density in the re-melted TBCs [43,44]. Moreover, preheating a substrate can cause a smaller energy loss due to a reduction in energy conduction into residual coatings and substrate, while the surface absorption of a laser energy increases with the enhancement of absorptivity [43,45]. Thus, more absorbed laser energy is used for melting the ceramic and achieving an increased depth of re-melted layer [43,45]. Therefore, in this study, to achieve a controllable and optimized segmented cracks, the method, referring to laser re-melting assisted by induction heating, is adopted to modify the as-sprayed TBCs, especially, a combination method of employing a low laser energy and a high preheating temperature is expected to avoid thermal damage of the underlying SX substrate.

## 2. Materials and Methods

### 2.1. Materials

**Figure 1:**
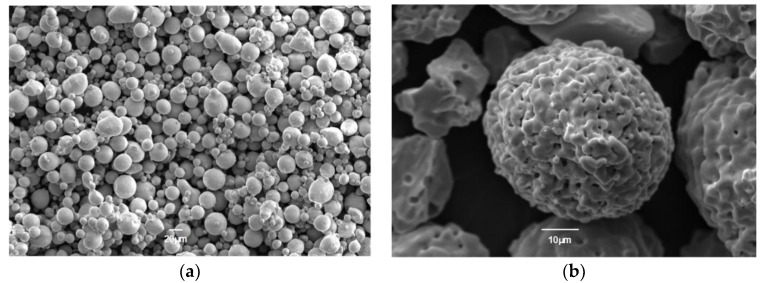
Morphologics of powders: (**a**) NiCoCrAlYTa and (**b**) 7YSZ.

A Ni-based SX superalloy (DD6, Ni–5.6Al–4.3Cr–9Co–2Mo–8W–7.5Ta–2Re–0.5Nb–0.1Hf–0.006C) was selected as the substrate. An intermetallic NiCoCrAlYTa powder (Amdry 997, Sulzer-Metco, New York, NY, USA) with a particle size of 15–45μm (Figure 1a) was used to prepare the bond coatings (BC). A partially yttria-stabilized powder (ZrO_2_-7 wt% Y_2_O_3_, AMPERIT 827, HC Starck, Goslar, Germany) was used to prepare the top coating (TC). The grain diameter of the powder was 10–45 μm (Figure 1b). The SX superalloy surface was grit-blasted with 24 mesh silicon carbide prior to spaying. Low temperature supersonic flame spraying (LT-HVOF) system (K2, GTV Verschleißschutz GmbH, Luckenbach, Germany), equipped with an IRB2400 ABB robot, and a GTV delta three anodes APS system (MF-P-1000, GTV, Verschleißschutz GmbH, Germany) was employed to spray the BC and TC respectively.

### 2.2. LSR Experiments

**Figure 2:**
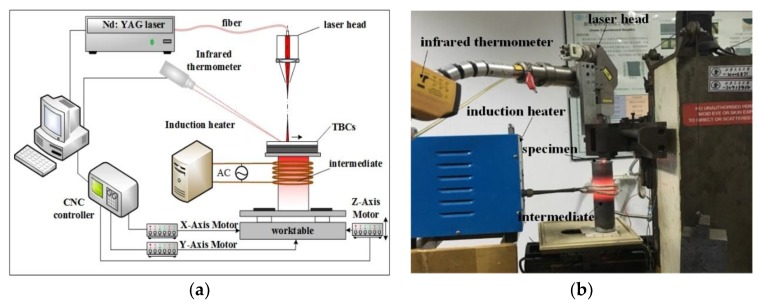
(**a**) Schematic diagram and (**b**) experimental setup of the LSR system.

Subsequently, the LSR experiments were performed as depicted in the schematic view (Figure 2a) and the experimental setup (Figure 2b). Prior to laser treatment, the induction heater was used to preheat the substrate to the preset temperature 800 °C and non-preheating for reference. A JK300D Nd: YAG pulsed laser (GSI, Birmingham, UK) was employed to scan perpendicular to the TCs surface. To achieve the minimum laser thermal damage, a combination of employing the high preheating temperature and adopting low laser energy was experimentally predetermined. These parameters were selected based on the following criteria: (a) surface re-melting capability and (b) formation of continuously compact re-melted layer. Energy values determined were 56.6 and 86 J/cm^2^ for preheating temperatures of 800 °C and room temperature respectively. The combination of low laser energy density (56.6 J/cm^2^) and non-preheating condition and the combination of high energy density (86 J/cm^2^) and preheating condition were also adopted for reference. The parameters used in this experiment are presented in Table 1. The experiments were conducted three times under the same processing parameters.

### 2.3. Characterization

After laser re-melting, in order to avoid the thermal damage to the segmented cracks in the laser re-melted layer during heat treatment, all the specimens were first metallurgically processed for obsevering the microstructure of re-melted coatings. Then, the specimens were mechanically polished up to a mirror finish, parall to the laser scanning track. After the microstructure observation, then all species were tubed in a silica glass tube under intert argon atmosphere and were subjected to heat treatment (1050 °C / 4 h, AC). A standard solution treatment (20 g CuSO_4_ + 100mL HCl + 80mL H_2_O solution) for a DD6 matrxi was seleceted to analyze the lognitudial microstructure of the alloy. The microstructures of the manufactured coatings were determined using scanning electron microscopy (SEM, JEOL, Tokyo, Japan) and energy dispersive spectroscopy (EDS, JEOL, Tokyo, Japan).

## 3. Results 

**Figure 3:**
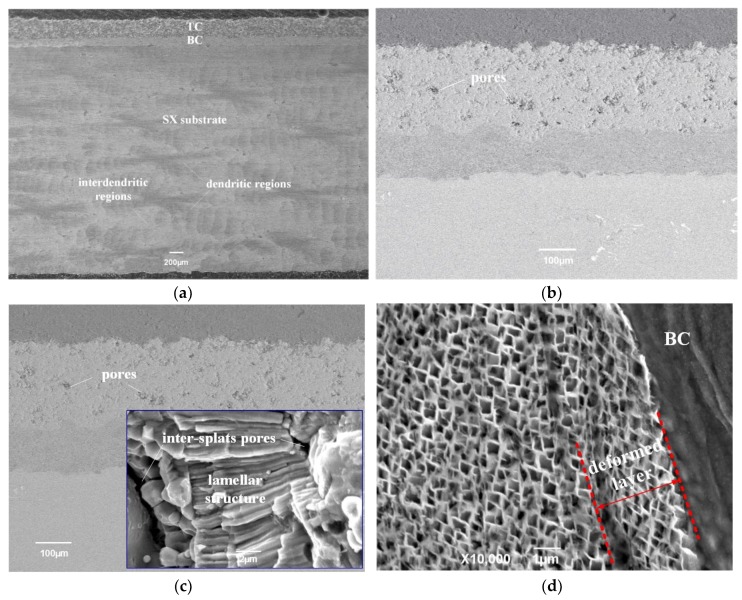
Secondary electron (SE) image (**a**) and (**b**) backscattering electron (BSE) enlarged image of TBCs/ SX superalloy system; (**c**) fracture microstructure of as-sprayed coating; (**d**) SX superalloy microstructure close to the interface.

Figure 3 shows the typical cross-sectional micrographics of as-sprayed TBCs coated on SX superalloy substrate. It was found that a TC with a thickness of about 250 ± 10 μm and a BC with a thickness of about 100 ± 10 μm are deposited on the SX superalloy substrate. Massive un-melted particles and large voids are discovered at the as-sprayed splats in the enlarged view of the coating, as shown in Figure 3b. As can be observed in Figure 3c, the fracture microstructure of the as-sprayed TBCs shows the typical lamellar structure and massive inter-splat pores and microstructure cracks, which is due to the random deposition and incomplete overlap of adjacent splats in the process of plasma spraying [46]. The close-up image in Figure 3d reveals the substrate microstructure close to the interface. It is obvious that the γ′ phase close to the interface between BC and SX superalloy substrate was regularly arranged and was severely distorted due to grit blasting. The depth of the deformed layer was approximately 2~3 μm.

**Figure 4:**
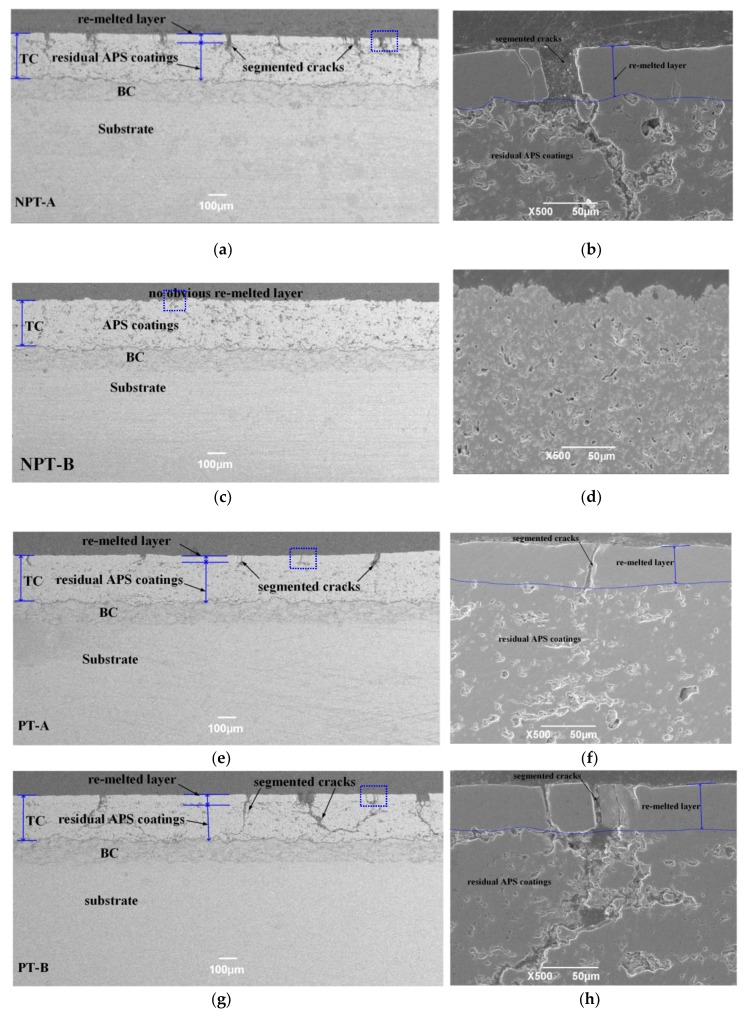
SEM images showing cross-sectional microstructures of (**a**,**b**) NPT-A, (**c**, **d**) NPT-B, (**e**,**f**) PT-A, and (**g**,**h**) PT-B samples, respectively.

Figure 4 shows the cross-sectional micrographics of re-melted coatings with different process parameters. For the NPT-A, PT-A and PT-B samples, a uniform and dense re-melted layer was formed after laser re-melting as shown in Figure 4a,e,g respectively. While for the NPT-B sample (Figure 4c,d), the lower laser energy density (56.6 J/cm^2^) had no ability to re-melt the coatings. By comparing the re-melted microstructure of the NPT-B sample and PT-A sample, it can be concluded that preheating can lower the laser energy threshold that is required for continuously re-melting the coating. For the three re-melted coatings, the average height of the re-melted layer was about 62, 52 and 78 μm for the NPT-A PT-A and PT-B samples respectively. In view of the segmented cracks, the NPT-A sample has severe crack distribution in terms of crack dimension and density, i.e., the maximum crack length reaches up to approximate 320 μm and the maximum crack width as high as approximately 50 μm. What is worse, certain longitudinal cracks propagate toward their direction deflection and develop a transverse crack along the residual as-sprayed coating, as shown in Figure 4a,b. Although the PT-B sample has lower segmented cracks density, it exhibits the most severe and damaged cracks in terms of the crack length and crack propagation in all re-melted coatings. For the PT-B sample, as shown in Figure 4g,h, the segmented cracks even extended to the interface of the bond-coat and top-coat of the PT-B sample and spread through the splat. What is worse, crack coalescence occurs between vertical cracks and horizontal cracks. While the segmented cracks of the PT-A sample have been improved in view of the dimension and density, it can be seen from Figure 4e and Figure 4f that the cracks are mostly distributed limited to the re-melted layer of the crack width as small as 10~20 μm. It can be concluded that combining the low laser energy and elevated preheating temperature is an effective means of lowering heat input and bringing about a smoother thermal gradient. Accordingly, the preheating substrate can remarkably minimize the cracking susceptibility of ceramic coating and develop a homogenous re-melted coating with a controllable segmented cracks distribution in laser re-melting APS TBC.

**Figure 5:**
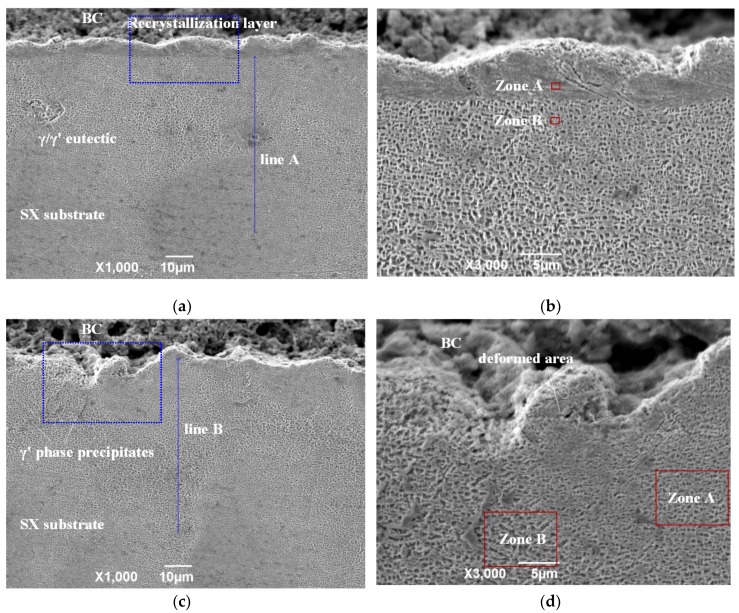

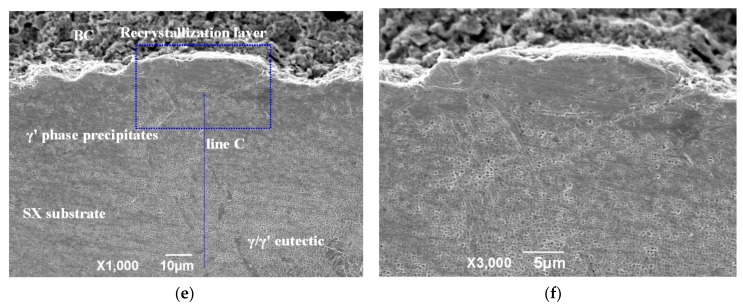
SX superalloy microstructure close to the interface of (**a**,**b**) NPT-A, (**c**,**d**) PT-A and (**e**,**f**) PT-B samples after heat treatment.

**Figure 6:**
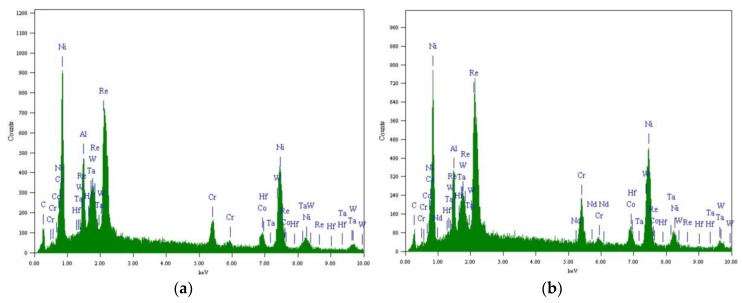
Element distribution of (**a**) domain A and (**b**) domain B in Figure 5b.

The close-up images in Figure 5 reveal the substrate microstructure of various samples after heat treatment. It can be seen that the microstructure in the top layer of substrate is different from the matrix microstructure, for both the NPT-A sample (Figure 5a,b) and PT-B sample (Figure 5e,f). The depth of the layer is about 5~6 μm and 6~8 μm for the NPT-A and PT-B sample, respectively. EDS results of zone A (as shown in Figure 6a) and zone B (as shown in Figure 6b) show that the two layers contain similar chemical composition distributions to the matrix composition. Thus, it can be concluded that the top layer is the surface recrystallization layer. Close to the surface recrystallization, the γ′ phase was slightly distorted and irregularly arranged in the NPT-A sample, as shown in Figure 5b. The size of the γ′ phase tends to be much larger, less uniform and irregularly shaped. It is indicated that γ′ phase coarsening can lead to degradation of the stress rupture properties of SX superalloy [47]. For the PT-B sample, the SX superalloy mainly consisted of a relatively small amount of γ′ phase while a certain γ′ phase dissolution occurs as shown in Figure 5e and Figure 5f. It has been reported that the γ′ phase dissolution plays a great role for grain recrystallization [48]. However, no clear evidence of surface recrystallization was revealed at the substrate microstructure of the PT-A sample (Figure 5c,d). Only some precipitated coarse and blocky γ′ phase was observed nearby the BC/SX superalloy interface. It has been reported that the coarse γ′ phase precipitates, and incoherent γ-γ′ interfaces are detrimental to the creep resistance of SX turbine blades [49,50]. In addition, some deformed zone, consisting of a seriously distorted certain γ′ phase, occurs nearby the interface in enlarged view as shown in Figure 5d. 

**Figure 7:**
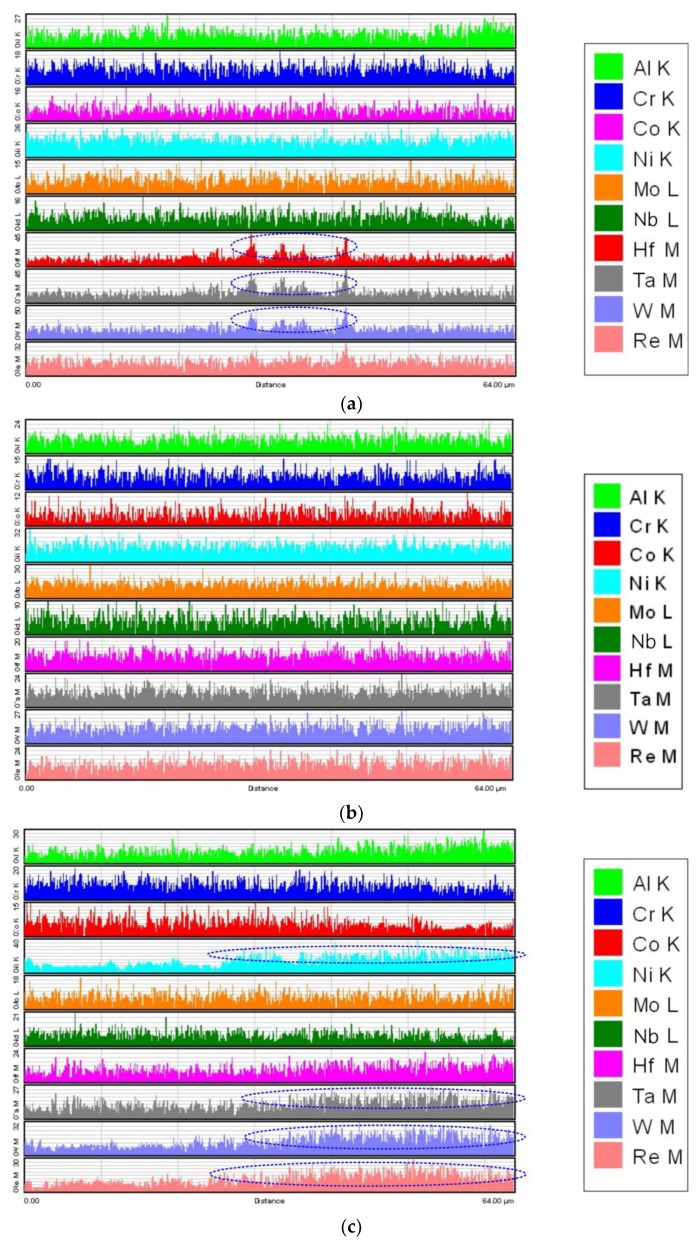
Element distribution along (**a**) line A, (**b**) line B and (**c**) line C scanning by EDS.

Figure 7a–c shows the element composition distribution of the various samples along line A, line B and line C in Figure 5, respectively. For the NPT-A sample, as shown in Figure 7a, it is indicated that the concentrations of Hf, Ta and W in the marked area are obviously higher than those in other regions. It can be concluded that the formed precipitations are more likely the MC carbide since Hf and Ta are primary forming elements of MC carbide [51]. However, the elements distribute uniformly and vary slightly at the different locations for the PT-A sample, as depicted in Figure 7b. Unlike the other samples, the PT-B sample exhibits an obvious tendency of diffusion of elements. A relatively high peak of Cr, Ni, W and Re have been detected close to the interface, while the concentrations of those elements are evidently low in the regions away from the interface for the PT-B sample, as shown in Figure 7c. It can be assumed that the diffusion of the γ′ forming elements (i.e., Ni, Ta, W, Re) is toward the interface under the effect of relatively high plastic strain and stored energy. The directional element diffusion in the PT-B sample is mainly due to the high temperature and high-level of thermal stresses, providing more driving force for elements moving beyond the lattice limit.

## 4. Discussion

### 4.1. Segmented Crack Behavior

**Figure 8:**
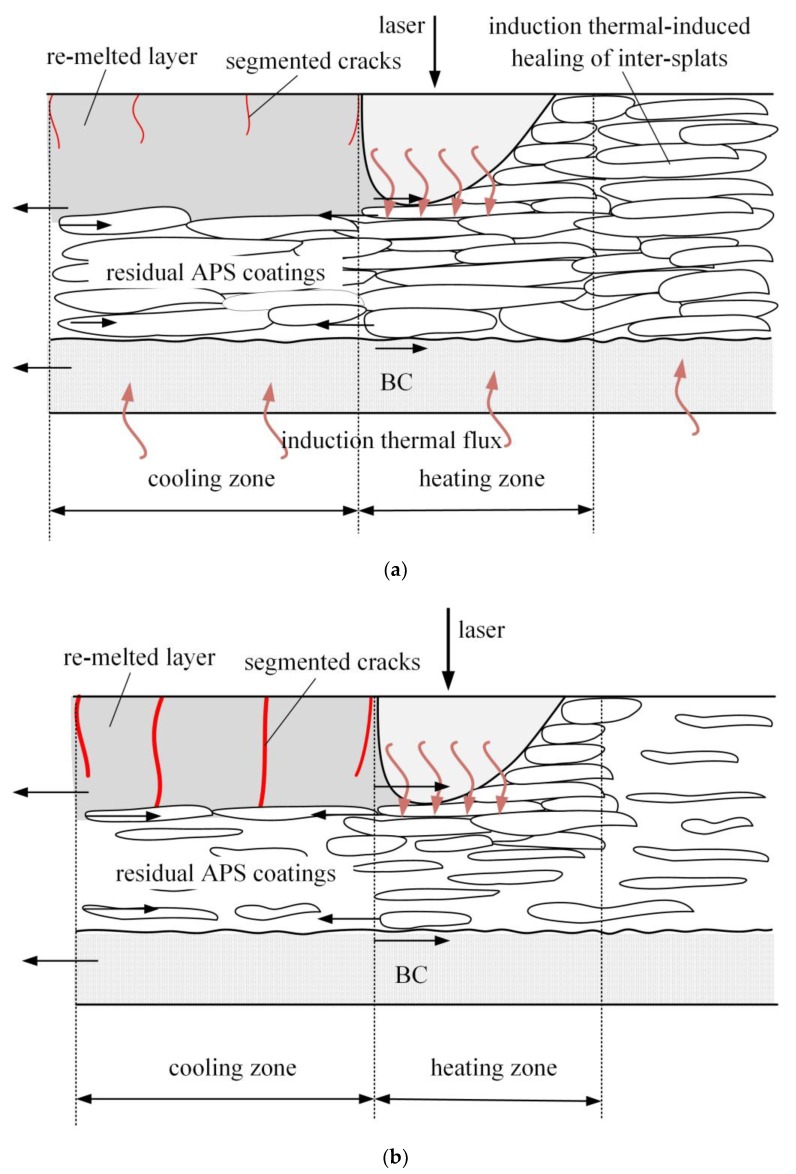
The schematic of microstructure evolution of (**a**) non-preheating sample and (**b**) preheating sample during the laser re-melting process.

The partial re-melted APS coatings consist of four layers as illustrated in Figure 8—the dense re-melted layer, the porous residual APS splats, the bonding coating and the substrate. The residual APS coatings remain the lamellar structure with massive inter-splats pores and interior voids. The as-sprayed coatings consist of a lamellar structure with massive inter-splat pores and interior voids, which have an approximate 50% reduction in in-plan elastic modulus with respect to the bulk materials [52,53,54,55]. While the laser re-melting can eliminate these pores to develop a dense re-melted layer, when heating or cooling, the four layers will suffer the thermal mismatch stress due to the inter-layer deformation constraints. As for the re-melted layer and the residual APS layer, when the laser moves away, the coatings system experiences a cooling shrinkage. While the re-melted layer is constrained by the surrounding APS splats that have relatively small strain as the inter-splat pores can act as a tolerance space [56], they will be subject to a tensile stress. Accordingly, for the non-preheating sample, the mismatch strain and corresponding tensile stress will initiate and spread the segmented cracks in the re-melted layer, resulting in severe segmented cracks distribution (Figure 4a,b).

It is reported that thermal exposure sintering can lead to healing of inter-splat pores and intra-splat cracks, which accordingly results in an increase of elastic modulus [57,58]. Therefore, for the preheating sample, as illustrated in Figure 8b, the significant sintering effect of preheating can heal the inter-splat pore and induce a decrease of strain difference between the re-melted layer and residual APS coating. Thus, for the PT-A sample, the healing of the inter-splat pore and low residual stress are the main reasons for improving segmented crack distribution. The results can further prove that laser re-melting with the assistance of a high degree of induction heating is an effective method for lowering residual stress level and developing a uniform dense modified coating. Although adopting the same preheating condition as the PT-A sample, the segmented cracks could not be effctively improved in the PT-B sample. This is mainly due to the fact that, on the one hand, for the Gaussian pulse laser, enhancing the laser peak power will increase irradiated energy in the laser spot center and makes the thermal gradient greater between the center and boundary of the laser track, resulting in an increase of residual stress in the re-melted layer. On the other hand, the combination of high laser energy and high preheating temperature can cause an overmuch energy input into the coatings, which can accordingly enhance thermal mismatch stress as well as the probability of initiating a crack. 

### 4.2. Recrystallization Behavior

Under extreme conditions, the ceramic coating and metallic matrix system with huge thermal mismatch stress is often subjected to complex interfacial strength and microstructure evolutions [21,22]. Mismatch deformation between TBCs and the substrate is induced by a pulsed laser and is changed during the rapid heating (duration of the pulse, i.e., 1 ms in this study) and rapid cooling process (interval between two neighboring pulses, i.e., 249 ms in this study). Thus, the TBCs/substrate interface is subject to a changing thermal mismatch stress shock, and plastic strains may occur in the interface. It is believed that the driving force for the SX superalloy recrystallization is related to the release of the residual stresses [35,40]. As a result, the residual stresses and stored energy provide the driving force for complex microstructure evolution to different degrees, i.e., element diffusion, lattices distortion, and γ′ phase dissolution, more probably resulting in significant SX recrystallization during the subsequent thermal treatment. For the PT-B sample, the combination of high laser energy (86 J/cm^2^) and elevated preheating temperature (800 °C) cause an overmuch energy input into the coatings, relatively large mismatch stresses, and more energy stored in the interface. Therefore, the most serious surface recrystallization, significant element directional diffusion towards interface, were found in the PT-B sample. The dissolution of the γ′ phase was also observed in the PT-B sample. One the one hand, as researches indicated that the residual stress can assist the dissolution of the γ′ phase into the γ matrix [59], thus, the great plastic strain and stored energy nearby the interface can drive the γ′ phase diffuse and dissolve into the matrix. On the other hand, the dissolution of the γ′ phase is more prone to occur at the elevated temperature, i.e., the temperature is closer to the γ′ phase solvus temperature ~1260 °C [48]). For the NPT-A sample, the combination of high laser energy (86 J/cm^2^) and non-preheating condition can also cause severe thermal damage to the interface of the TBCs/SX matrix and introduce the surface recrystallization of SX superalloy substrate. 

**Figure 9:**
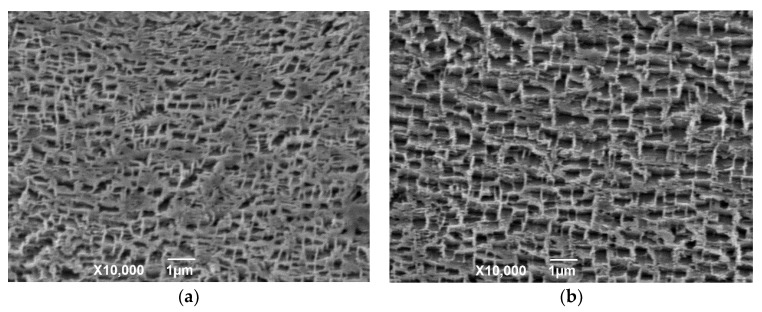
Detailed view of (**a**) domain A and (**b**) domain B in Figure 5d.

Encouragingly, the PT-A sample exhibits the microstructure with no evident surface recrystallization. It is proved that laser re-melting adopting a low laser energy (56.6 J/cm^2^) with the assistance of high-level induction preheating (800 °C) can significantly reduce thermal transferring into the SX matrix, thereby avoiding the surface recrystallization of the underlying SX matrix. Furthermore, the uniform thermal evolution ultimately weakens the thermal impact and stress shock to the interface during each laser pulse action. This can lead to a significant reduction in accumulated residual stresses and develop a relatively stable SX microstructure. However, the PT-A sample still exhibits certain coarse γ′ phase precipitations (as shown in Figure 5c,d) and γ′ phase with various sizes and different degrees of distortion at various zones nearby the interface. Figure 9a,b shows the detailed view of zone A and zone B in Figure 5d respectively. It is indicated that the γ′ phase in zone A was severely distorted and has a relatively small size, while the γ′ phase in zone B was obviously large and cubic-shaped. Unlike the NPT-A and PT-B sample (the interface stored energy is mainly used for driving surface recrystallization), the stored energy and plastic strain still remain at the interface for the PT-A sample. It has been said that the morphology of the γ′ phase is dominated by the elastic strain energy and interfacial energy between γ matrix and γ′ precipitates [51]. For the PT-A sample, the γ′ phase begins to grow during heat treatment and then the γ′ cuboid starts to split into several smaller ones to release the elastic strain energy when γ′ phase grows to a critical size [60,61]. Therefore, the γ′ phase tends to be small at the high strain energy zone and be relatively large at the low strain energy zone in the PT-A sample. As the thermodynamic driving force releases, meanwhile, the γ matrix channel tends to decrease and the secondary γ′ phase is nucleated again; as a result, some γ′ phase precipitates from the γ matrix channel to form coarse γ′ phase precipitates as shown in Figure 5c,d. Nevertheless, evolution behavior of the γ′ phase and segmented cracks under high temperature thermal shock and their effect on the interfacial strength of these modified APS TBCs should be further investigated in future work.

## 5. Conclusions

A laser assisting with induction heating was adopted to re-melt the APS-TBCs/SX matrix multilayer system, aiming to minimize the thermal damage of this system. Four sets of parameters were used in the experiment: the combination of high laser energy (86 J/cm^2^) and non-preheating (NPT-A); combination of low laser energy (56.6 J/cm^2^) and non-preheating (NPT-B); combination of low laser energy (56.6 J/cm^2^) and elevated preheating temperature (800 °C) (PT-A); and the combination of high laser energy (86 J/cm^2^) and elevated preheating temperature (800 °C) (PT-B). The following conclusions can be drawn.

- Although they have the same laser energy, a continuous re-melted layer formed in the PT-A sample while there was no sign of melting in the NPT-B sample. It can conclude that preheating can lower the laser energy threshold that is required for continuously re-melting the coating.
- Both lowering laser energy and increasing the preheating temperature can result in a decrease of thermal mismatch strain between the re-melted layer and residual as-sprayed layer, resulting in a relatively low-level residual stress in the re-melted layer. The PT-A sample has the less severe segmented cracks compared to the NPT-A and PT-B sample in terms of crack dimension and crack density.
- Surface recrystallization occurs at both NPT-A and PT-B samples, while only certain distorted γ′ phases and some coarse precipitated γ′ phases were found nearby the interface between the BC and SX matrix for the PT-A sample. The remarkably reduced thermal accumulation and stain stored energy close to the interface play a crucial role in developing the microstructure with no evident surface recrystallization.

## Figures and Tables

**Table 1:** Experimental parameters of LSR.

Label	Preheating Temperature (°C)	Pulse Energy Density (J/cm^2^)	PULSE Width (ms)	Spot Diameter (mm)	Laser Frequency (Hz)	Laser Scanning (mm s^−1^)
**NPT-A**	Room Temperature	86	1	4	40	10
**NPT-B**	Room Temperature	56.6				
**PT-A**	800	56.6				
**PT-B**	800	86				
